# Medical Opinion and Movement

**Published:** 1910-07-23

**Authors:** 


### Medical Opinion and Movement.

### Fifty Hours' Delay between Births of Twins.

Dr. C. J. Hill-Aitken publishes in the *Transvaal Medical Journal* an interesting case in which natural delivery of the second of twins occurred after a fifty-hours' delay. A native woman was delivered of a child at 12 noon on a Thursday. After the birth, labour pains ceased and did not return. The relatives waited patiently until the following afternoon, and then decided to seek European medical advice. The author was sent for, and was told that while active interference was, if possible, to be avoided, his advice would be welcomed. Reaching his patient about 7.30 p.m. the same evening, he found that while no labour pains were complained of the uterus was active, as it could be felt alternately hardening and softening under the hand. The child was alive and active in its movements. Internal examination showed that there was no hæmorrhage from the uterus or from the cord of the first child, that the bag of membranes was unbroken, and that the head of the child was lying at the pelvic brim, which was wide and unobstructed. On explaining these facts to the relatives, the author was pleased to find that they were willing to await the onset of the second labour, as he did not wish to apply high forceps or to perform version in a native hut with scanty hot-water supply and no table on which to place his patient. He also preferred not to take the risk of rupturing the membranes, although such a proceeding might have hastened matters. After putting the patient on drachm doses of elixir aletris, four-hourly, and recommending the frequent cleansing of the external genitalia, he left. Labour set in the following morning at 4.30 a.m., and the second child was born at 11 p.m. There was thus an interval of fifty-one hours between the birth of the first and of the second child. The author considers that the case may be considered one of natural delivery despite the administration of aletris, for the womb was by no means inactive when examined. Both mother and children were well ten days after delivery.

### Prognosis in Cancer of the Mouth.

There is no doubt that in respect of cancer of the tongue, tonsils, and buccal cavity generally, the results of operative treatment are still very often disappointing; and that complete and permanent cure follows much less often than in other regions of the body. Some time ago Mr. Butlin published the results of his own practice in this connection, and an abstract of his conclusions was given in The Hospital (January 16, 1909, p. 396). Now three American surgeons publish the statistics of the results obtained in these cases at the Massachusetts General Hospital, and the comparison of their conclusions with those of the President of the Royal College of Surgeons is of interest. Sixty-two patients were operated on for cancer of the tongue and floor of the mouth; the mortality within two months of operation was 12.9 per cent.; the percentage of those who survived three years was 17.2. Of the latter one had a local recurrence seven years later, but after removal of this has now been free for a further five years. Curiously, the duration of the growth before removal seems to have little influence; the average was actually longer among the successful than the unsuccessful cases. On the question of dissecting out the cervical glands the American surgeons are pessimistic. They say this practice is attended by very high mortality and an extremely low percentage of cures. Early diagnosis, prompt intervention, and widespread local removal are the means advocated for the improvement of the prognosis for these serious cases. Another American author, Dr. J. C. Warren, has dealt exhaustively with this subject. His conclusions, which were abstracted in this column on November 21, 1908, are more in accordance with those of Mr. Butlin than with those of the Massachusetts surgeons.

### Treatment of Joint Tuberculosis.

Dr. Ely writes on the subject of tuberculosis of the joints in *Surgery, Gynæcology, and Obstetrics.* He believes that only two of the articular tissues are subject to tuberculosis, the synovial membrane and the red marrow. The presence of each of these is dependent on function; when the joint has been deprived of function they disappear, and the disease then dies out. Therefore, to cure a tuberculous joint it must be deprived of function; but further operative resections than suffice to bring this about are unnecessary. In the knee he simply opens the joint and removes enough bone to secure ankylosis, paying no attention to the tuberculous tissue left behind, which will shrink and disappear when the joint has been destroyed; to remove two inches of bone is quite unjustifiable. Similarly in the hip; it is impossible to eradicate all the tuberculous tissue; so he declines to sacrifice the head, neck, and trochanter. Curetting a tuberculous ankle or tarsus is another common proceeding which is strongly condemned. In children conservative measures will often suffice for a cure in accordance with this theory, but in adults more reliance is to be placed upon eradication of the joint.

### Gonorrhœa in Children.

Hamilton, in the *Journal* of the American Medical Association, calls attention to the alarming increase in New York of gonorrhœa in children. He analyses the statistics of the Vanderbilt clinic in that city and finds that out of 344 cases observed the average age was 5 years. In 40 cases in which the upper part of the vagina was tested gonococci occurred in every one. Children would seem to be remarkably free from systematic infection, for no cases of cystitis, urethritis, and pelvic or general peritonitis were discovered. Inguinal adenitis occurred in two and arthritis in three cases. Four cases of ophthalmia from auto-infection occurred, and in 10 children nocturnal enuresis was induced which had previously not existed. Treatment consisted in irrigation and the use of vaccines. In most cases, except in children under six months old, treatment was started by an injection of 50 million gonococci; the injections were repeated every fifth day, the dose each time being increased by 10 million until five doses had been given, when an interval of 10 days was allowed to elapse before giving the sixth dose. It was usually unnecessary to give more than six doses, but in obstinate cases treatment was continued until 200 million gonococci were injected at a time. The author concludes that vaccine therapy has a place in the treatment of infantile gonorrhœa, because of the rapidity with which it brings about a cure in 85 per cent. of cases, and because of the ease of administration. It is harmless under proper conditions, and it is not necessary to take the opsonic index of the child's blood since it has no relation to the amount of the discharge. The use of vaccines eliminates the necessity of irrigations, which at times are conducive to masturbation, and may produce injury, however careful the treatment.

### Chamois-leather to Prevent Bedsores.

Dr. Morichau-Beauchant has published in the *Archives Médico-chirurgicales* a note concerning a procedure which, he thinks, may have great results in practice. In addition to the powders, cotton-wool, indiarubber cushions, etc., which ordinarily serve in the prevention of bedsores, he proposes to interpose between the sheets and the points of contact with them of the body a piece of chamois-leather. The article which in commerce goes by that name is nothing more or less than a sheepskin which has undergone a particular preparation called "chamoisage." It is the soft side of the skin which should be applied to the part which it is desired to protect. Being of great firmness, it can be used in lifting the patient. It washes well, is durable, and comparatively inexpensive. It can be used by itself or may be interposed between a rubber air-cushion or water-bed. The author was struck by the success which attended its use in a recent case in which the ordinary methods had been tried without benefit. While little known in France, he believes that this method is largely used on the Continent, for a manufacturer of Niort declared to him that he exported annually large numbers to Continental hospitals.

### Effect of Fat Ingestion on Mother's Milk.

In the *Rivista di Clinica Pedriatica* Dr. Malagodi discusses the effects on the fats of human milk caused by changes in the amount of the fats in the mother's diet. Since great differences of opinion would seem to exist among authorities on the point, he made investigations in the obstetric clinic at Bologna. The milk in each case was carefully analysed before and after the change in diet. In only one case was he able to detect an increase in the milk-fats after an increase of fat in the diet. On the other hand, a decrease of fat in the food gave a decrease of fat in the milk. An important change was also noted in the quality of the milk, which was reflected in an alteration in the colour and density of the milk-fat. The author concludes that some attacks of dyspepsia occurring in breast-fed children can be accounted for by changes in the food of the mother.

### A New Suture.

Dr. Kermisson recently reported to the Paris Academy of Medicine on the work of Dr. Chastenet de Gery respecting the utility of a new absorbable suture made from fish-skin. The new suture, while absorbable and aseptic, has the advantage over catgut that it is made from material which never contains tetanus bacilli. Dr. Kermisson, after a preliminary trial on animals, used the material in fifteen operations of different kinds in man. In all cases absorption was complete and asepsis ensured. The sutures are exceedingly strong, but are less elastic than catgut, and have the disadvantage of always being of a uniform size. If this last objection can be overcome the material promises to be of surgical value.

### Stone in the Urethra.

Professor Charles Morton reports, in the *Bristol Medical Journal*, a case in which a stone formed in the bladder around a piece of wood introduced into the urethra twenty-six years before. At that time the patient had had retention of urine, and in order to relieve himself he passed a kind of bougie, which he made of wood. The end of this broke off in his urethra, but, curiously enough, it relieved the retention. The broken-off end of the wooden instrument remained, however, in the urethra. He has at times since then had difficulty with micturition, for which, when marked, he used to pass a No. 4 metal catheter. In doing this he declared he struck the wood, which really was the stone. A few weeks before coming under observation a sinus formed in the perineum, and Dr. Peake, his medical adviser, had opened an abscess in that neighbourhood a day or two before the author saw him in consultation. A mass of induration was noticed more to the right than to the left of the perineum. The sinus and the abscess opening were in this mass, and pus and urine flowed from both. With considerable difficulty a No. 3 catheter was introduced into the bladder, and in passing grated upon a stone. The urine was purulent, but not offensive. A few days later the stone was removed. The mass of induration was very largely due to the size of the stone, which lay rather to one side of a pouched urethra. On cutting open the stone a piece of wood was found to form its nucleus. The cut surface of the stone measured approximately at its widest and longest parts one inch by two.

### Radium Therapy.

An interesting article appears in *Le Progrès Médical* by Dr. L. Chevrier on the treatment of open wounds and ulcers by means of small quantities of radium. According to this author, the effect of the radium shows itself in a marked manner by the rapid promotion of granulation and skin formation. He has carried out the treatment by injecting small doses of an insoluble salt of radium, such as radium sulphate, the amount injected being 20 to 60 microgrammes of the salt. More recently, however, he has had prepared for him ointments and powders containing minute quantities of the same radium salt, and these are spread over the wound or ulcer. The ointment used may be either plain vaseline or lanoline, or a mixture of both, or if an antiseptic effect is desired at the same time erythrol ointment may be used. One gramme of the ointment should contain 1 to 10 microgrammes of radium sulphate. In the case of the powders, an inert powder, such as powdered charcoal, or erythrol, sodium biborate, sodium bicarbonate, or sugar, may be used with 1 to 5 microgrammes of radium sulphate. As a result of these applications there is an abundant serous secretion from the wounds which the author compares to the lymphorrhœa which occurs after fulguration, and this secretion gives to the dressings a brownish, sometimes almost black, stain, the nature of which he is quite unable to explain. The author has treated a large number of cases in this way, and reports details of several in which deep wounds or indolent ulcers have quickly responded by healthy granulation and rapid skin formation. In some cases in which the raw surface was large the radium application has only been placed upon part of the surface, and the contrast in the healing processes of the different parts of the wounds has been most striking. These radium applications appear, therefore, to possess valuable healing properties. Dr. Chevrier obtains his preparations from a M. Jaboin, of Paris, but does not refer to the practical question of their costliness. Presumably the price would be prohibitive for everyday use.

### The Abuse of Stimulants.

More than once the voice of warning has been raised against a too meddlesome and busy method of therapy in the treatment of post-operative shock. This time it is Dr. H. G. Wetherill, Professor of Gynæcology and Surgery at Denver. He pleads against the abuse of all kinds of diffusible stimulants —hypodermic injections of ether, caffein, camphorated oil, strychnine, and the like, by which the patients are tormented with a view to combat shock, and in his opinion not infrequently poisoned. He suggests that the administration of these toxic drugs in excessive doses, in conditions in which elimination is retarded and difficult, leads to their accumulation in the system and may seriously compromise the recovery of the patient. Their action is chiefly on the nerve centres, which they temporarily excite, but soon afterwards leave them in a still more exhausted condition. Crile has shown by experiments on animals in a condition of profound shock that those treated with strychnine injections die much more quickly than the others, or if they recover their convalescence is much more prolonged. Blind credit should not be given to this emergency medication, and more restraint should be shown from the traditional desire "to do something." Put the patient to bed in the best possible position, keep him warm, let him absorb as much fluid as possible either by the mouth, rectum, subcutaneous tissue, or peritoneal cavity, and *let him alone*; that is the sum total of his advice. If the operation has been especially severe the pulse is feeble, and the condition of shock serious, saline infusions are of all ordinary remedies the most efficacious. As a last resource the author puts his faith in intravenous or subcutaneous injections of artificial serum as the most certain, most physiological, and least harmful means of recuperation.

### Cotton-wool and Inflammatory Tumours.

At a meeting of the Académie de Médecine Dr. Reynier recorded an interesting case of a breast tumour which was found to be of an inflammatory nature and caused by the absorption into the lymphatics of filaments of cotton-wool used as a dressing over an ulcerated surface. The case was that of a woman aged forty-one who had a large ulcerated tumour of the right breast. It was hard at certain points and fluctuated at others; it was not at all adherent to the deep tissue. It had been kept covered by the patient with a layer of cotton-wool. In the axilla were two or three glands, which, according to the patient, preceded the ulceration of the tumour. A diagnosis of cystic adenoma was made in process of malignant degeneration. The tumour was removed, and examination showed it to be a sarcoma grafted upon an adenofibroma. A little while after the operation a hard bossy mass appeared adherent to the lower margin of the pectoralis major muscle. This was also removed, and on microscopic examination it was found to consist of fibrous tissue, poor in cells, enveloping the muscular fibres, and scattered among the fibrous tissue were groups of multinuclear cells. These cells were in the lymphatic capillaries and their presence was caused by filaments of cotton embolised in the vessels. This second tumour was therefore purely inflammatory and due to the penetration of cotton fibres into the lymphatics from the cotton-wool with which the ulcerated surface had been dressed. The author had met with two other similar cases.

### Lumbar Injections for Tabes Dorsalis.

At a meeting of the Société de Biologie Dr. Sicard explained his method of treating certain cases of tabes dorsalis, in which the symptoms are limited to the lower part of the body—lightning pains of the lower limbs, bladder crises, sphincter troubles, etc. The object of the treatment is to induce by means of lumbar injections of saline solution a local meningeal reaction sufficient to excite the medullo-radiculo-ganglionic vascularisation and to liberate in part at least the nerve roots and lepto-meningeal culs-de-sac from the embryonic infiltrates which surround and press upon them. For this purpose he makes intra-spinal injections of three or four cubic centimetres of saline solution 8 per 1,000 every week or fortnight. He has employed the method in fourteen cases and claims to have obtained remarkable improvements in the symptoms as a result of the treatment. He advocates mercurial treatment at the same time and thinks that the injections facilitate the local effect of the mercury introduced into the general system.

